# Extent of Oral–Gut Transmission of Bacterial and Fungal Microbiota in Healthy Chinese Adults

**DOI:** 10.1128/spectrum.02814-22

**Published:** 2023-01-10

**Authors:** Man Kit Cheung, Sylvia L. Y. Tong, Martin C. S. Wong, Jason Y. K. Chan, Margaret Ip, Mamie Hui, Christopher K. C. Lai, Rita W. Y. Ng, Wendy C. S. Ho, Apple C. M. Yeung, Paul K. S. Chan, Zigui Chen

**Affiliations:** a Department of Microbiology, Faculty of Medicine, The Chinese University of Hong Kong, Hong Kong Special Administrative Region, People’s Republic of China; b Centre for Gut Microbiota Research, The Chinese University of Hong Kong, Hong Kong Special Administrative Region, People’s Republic of China; c Jockey Club School of Public Health and Primary Care, The Chinese University of Hong Kong, Hong Kong Special Administrative Region, People’s Republic of China; d Department of Otorhinolaryngology, Head and Neck Surgery, Faculty of Medicine, The Chinese University of Hong Kong, Hong Kong Special Administrative Region, People’s Republic of China; e Stanley Ho Centre for Emerging Infectious Diseases, Faculty of Medicine, The Chinese University of Hong Kong, Hong Kong Special Administrative Region, People’s Republic of China; Jilin University

**Keywords:** oral–gut microbiota axis, 16S, ITS1

## Abstract

Recent studies have provided evidence on the presence of an oral–gut microbiota axis in gastrointestinal diseases; however, whether a similar axis exists in healthy individuals is still in debate. Here, we characterized the bacterial and fungal microbiomes in paired oral rinse and stool samples collected from 470 healthy Chinese adults by sequencing the 16S rRNA V3–V4 and ITS1 regions, respectively. We hypothesized that there is limited oral–gut transmission of both the bacterial and fungal microbiota in healthy Chinese adults. Our results showed that the oral and gut microbiota in healthy individuals differed in taxonomic composition, alpha and beta diversity, metabolic potential, and network properties. Bayesian analysis showed that the vast majority of subjects had negligible or low bacterial and fungal oral-to-stool contribution. Detailed examination of the prevalent amplicon sequence variants (ASVs) also revealed limited cases of sharing between the oral and stool samples within the same individuals, except a few bacterial and fungal ASVs. Association analysis showed that sharing of the potentially transmissible fungal ASVs was associated with host factors, including an older age and a higher body mass index. Our findings indicate that oral–gut transmission of both bacterial and fungal microbiota in healthy adults is limited. Detection of a large amount of shared bacterial or fungal members between the oral and gut microbiome of an individual may indicate medical conditions that warrant detailed checkup.

**IMPORTANCE** The oral–gut microbiota axis in health is a fundamentally important and clinically relevant topic; however, our current understanding of it remains biased and incomplete. By characterizing the bacterial and fungal microbiomes in paired oral rinse and stool samples from a large cohort of healthy Chinese adults, here we provided new evidence that oral–gut microbiota transmission is limited in non-Western population and across biological domains. Our study has established an important baseline of a healthy oral–gut microbiota axis, with which other disease conditions can be compared. Besides, our findings have practical implications that detection of a large amount of shared bacterial or fungal members between the oral cavity and gut within the same individual as an indicator of potential medical conditions.

## INTRODUCTION

The oral cavity and gut represent the two largest microbial ecosystems in the human body. Recent studies have provided evidence on the presence of an oral–gut bacterial microbiota axis in gastrointestinal diseases, including colorectal cancer and colitis (1, 2). However, transmission of oral microbes to the gut in healthy individuals is traditionally believed to be limited due to the presence of multiple barriers such as gastric acid and bile acids (3). To date, multiple studies have attempted to elucidate the extent of oral–gut microbiota transmission in health; however, contradictory results have been reported. While some studies have provided evidence on the seeding of oral bacterial populations in the gut or an extensive oral–gut microbiota transmission, others have reported limited overlaps of the microbiota between the two body sites (4–8). Unfortunately, these studies suffer from a limited taxonomic resolution, a small sample size, or the use of computational pipelines with unjustified assumptions (8). Besides, all these studies were based on Western populations and the majority of them focused only on the bacterial microbiota. As a result, our current understanding on this fundamentally important and clinically relevant topic remains biased and incomplete.

In this study, we characterized the bacterial and fungal microbiota in paired oral rinse and stool samples from a large cohort of healthy Chinese adults (*n* = 470) by sequencing the 16S rRNA V3–V4 and internal transcribed spacer 1 (ITS1) regions, respectively. We then examined the extent of oral–gut microbiota transmission by using a Bayesian approach and detailed comparisons within the same individuals at the amplicon sequence variant (ASV) level, which allows single-nucleotide resolution (9, 10). The aim of our work was to characterize the extent of oral–gut transmission of the bacterial and fungal microbiota at the finest taxonomic resolution within the same individuals based on a large Chinese population. Here, we defined oral–gut transmission of a microbe to be its translocation from the oral cavity to, and subsequent colonization of, the gut within the same individual (5, 8). We hypothesized that there is limited oral–gut transmission of both the bacterial and fungal microbiota in healthy adults.

## RESULTS

### Characteristics of the study cohort and sequencing data.

A total of 470 healthy Chinese adults with paired oral rinse and stool samples were included in the current analysis. The cohort consisted of 222 males and 248 females, with a mean age of 46 yrs (SD: 16 yrs) and a mean body mass index (BMI) of 23.0 (SD: 3.4) (Table 1). Other main characteristics of the study cohort are provided in Table 1. After quality filtering of the sequence reads, 470 and 370 subjects remained in the bacterial and fungal microbiome analysis, respectively. The subset used for fungal microbiome analysis shared similar characteristics with the full cohort (Table S1).

**TABLE 1:** Main characteristics of the study cohort (*n* = 470)^*a*^

Variable	Options	Count (%)
Sex	Male	222 (47.2)
	Female	248 (52.8)
Age, yr, mean (SD)		46 (16)
BMI, mean (SD)		23.0 (3.4)
Delivery mode	Vaginal	376 (80.0)
	C-section	36 (7.7)
	Unknown	55 (11.7)
	NA	3 (0.6)
Smoking	Yes	28 (6.0)
	No	442 (94.0)
Alcohol drinking	Yes	274 (58.3)
	No	196 (41.7)
Moderate–vigorous exercise	Yes	309 (65.7)
	No	161 (34.3)
Meat preference	Yes	216 (46.0)
	No	254 (54.0)
Vegetable preference	Yes	313 (66.6)
	No	157 (33.4)
Carbohydrate preference	Yes	261 (55.5)
	No	209 (44.5)
Supplement habit	Yes	146 (31.1)
	No	324 (68.9)
Bristol stool scale	Type 1–2	26 (5.5)
	Type 3–4	343 (73.0)
	Type 5–7	101 (21.5)

a SD, standard deviation; BMI, body mass index; NA, missing value.

Rarefaction curve analysis showed that at a sequencing depth of 1,000 and 200 reads, the majority of the bacterial and fungal diversity was captured, respectively (Fig. S1). Besides, highly consistent results between technical duplicates and between sequenced DNA standards and their theoretical composition, as well as a negligible number of reads generated in the negative control samples indicated a high quality and credibility of the data generated in this work (Fig. S2).

### Oral and gut bacterial, but not fungal, microbiota in health are distinct in composition.

**FIG 1:**
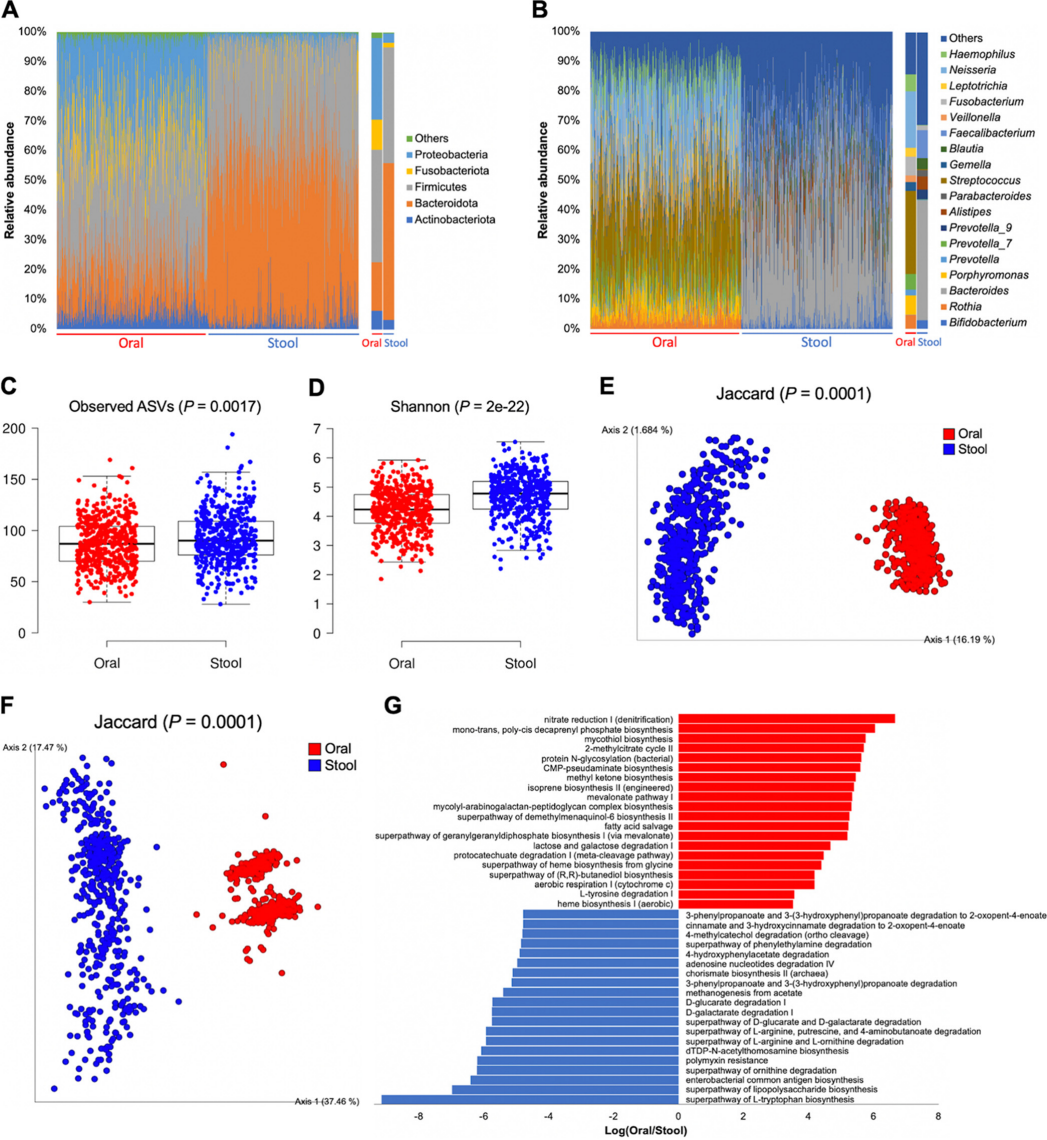
The bacterial microbiota in oral rinse and stool samples of healthy Chinese adults differ in taxonomic composition, alpha and beta diversity, and functional potential. (A, B) Taxonomic barplots of major bacterial phyla (A) and genera (B) for individual subjects (*n* = 470) (left panels) and their averages (right panels). Taxa with a mean relative abundance < 1% were grouped into “Others.” (C, D) Boxplots of the observed number of amplicon sequence variants (ASVs) (C) and Shannon diversity (D). (E, F) Principal coordinates analysis (PCoA) plots of the occurrence (presence/absence) of ASVs (E) and predicted MetaCyc pathways (F) based on Jaccard distance. (G) Top 20 differentially abundant MetaCyc pathways in the oral (red bars) and gut (blue bars) microbiomes as detected using Songbird. Alpha diversity was compared between groups using Kruskal–Wallis test, whereas PERMANOVA was used for testing of beta diversity.

The oral bacterial microbiota of our cohort was dominated by the phyla Firmicutes (mean relative abundance: 37.9%), Proteobacteria (27.5%) and Bacteroidota (16.3%) and the genera Streptococcus (28.1%), *Neisseria* (19.1%), *Porphyromonas* (6.6%), and *Fusobacterium* (6.4%) (Fig. 1A, B, and Data set S1). In contrast, the fecal bacterial microbiota was dominated by the phyla Bacteroidota (52.7%) and Firmicutes (39.0%) and the genera *Bacteroides* (40.5%) and *Faecalibacterium* (9.4%).

**FIG 2:**
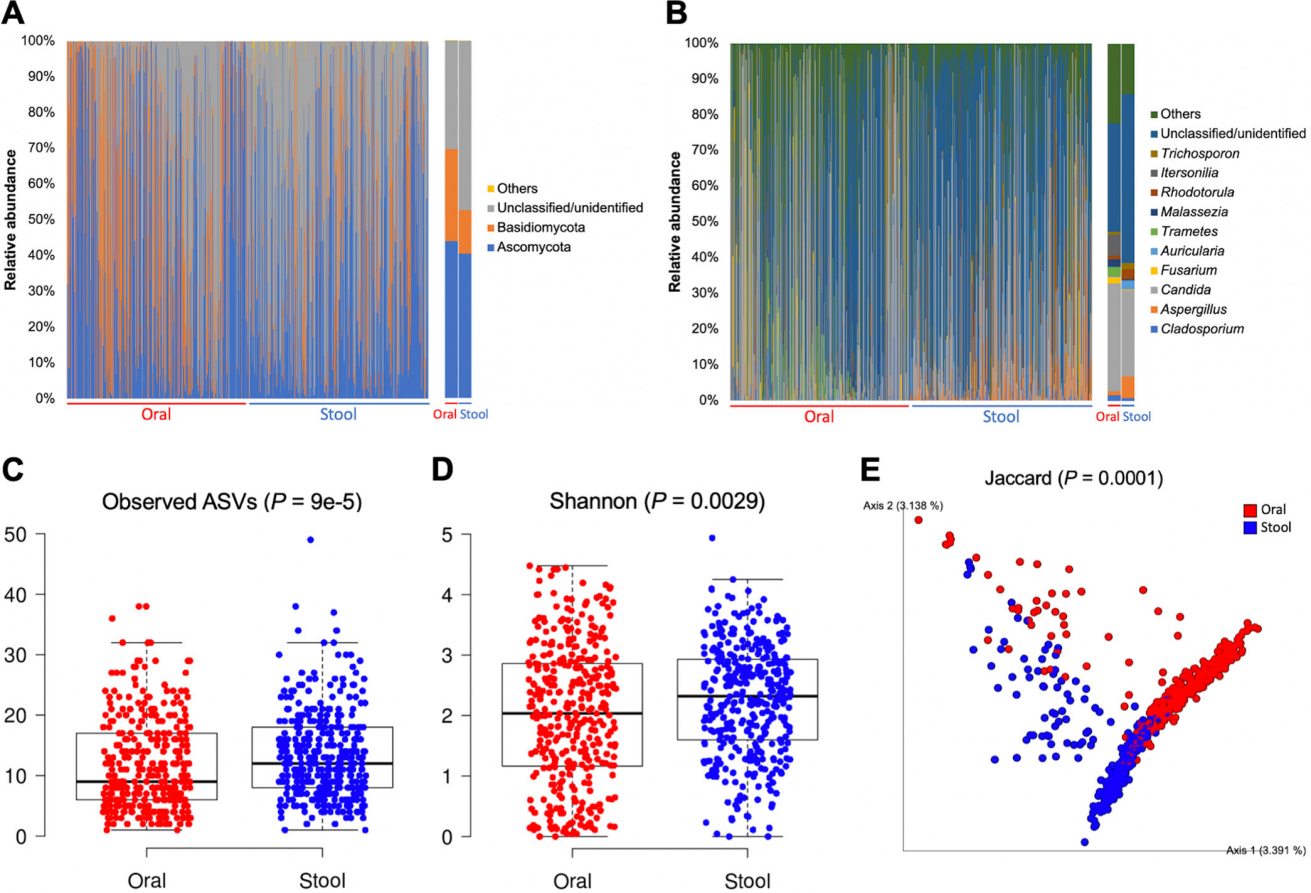
The fungal microbiota in oral rinse and stool samples of healthy Chinese adults differ in taxonomic composition and alpha and beta diversity. (A, B) Taxonomic barplots of major fungal phyla (A) and genera (B) for individual subjects (*n* = 370) (left panels) and their averages (right panels). Taxa with a mean relative abundance < 1% were grouped into “Others.” (C, D) Boxplots of the observed number of ASVs (C) and Shannon diversity (D). (E) PCoA plot of the occurrence of ASVs based on Jaccard distance. Alpha diversity was compared between groups using Kruskal–Wallis test, whereas PERMANOVA was used for testing of beta diversity.

Unlike the case of the bacterial microbiota, both the oral and fecal fungal microbiota of our cohort were dominated by the phyla Ascomycota (oral: 43.9%; stool: 40.4%) and Basidiomycota (oral: 25.7%; stool: 12.1%) (Fig. 2A and Data set S2). At the genus level, *Candida* was the predominant member in both body sites (oral: 30.3%; stool: 24.1%), and *Itersonilia* (oral: 5.9%; stool: 0%) and Aspergillus (oral: 1.1%; stool: 6.0%) were the next most abundant genera in the oral and stool samples, respectively (Fig. 2B).

### Oral and gut bacterial and fungal microbiota in health differ in alpha and beta diversity.

For both the bacterial and fungal microbiota, ASV richness and Shannon diversity significantly differed between the oral and stool samples (*P < *0.01) (Fig. 1C, D, 2C, and 2D). Beta diversity analysis based on the presence/absence of ASVs also revealed significant differences in the overall community structure of both the bacterial and fungal microbiota between oral and stool samples (*P = *0.0001) (Fig. 1E and 2E). Nonetheless, effect size analysis showed that the top host variables affecting the beta diversity of the bacterial and fungal microbiota were generally similar between oral and stool samples (Fig. S3).

### Oral and gut bacterial microbiota in health have different metabolic potential and network properties.

A principal coordinates analysis (PCoA) plot based on the predicted MetaCyc pathways revealed a distinct metabolic potential between the oral and fecal microbiota (*P = *0.0001) (Fig. 1F). Subsequent differential abundance analysis showed that the oral microbiome was most enriched in pathways of biosynthesis (11 out of top 20), whereas the fecal microbiome was most enriched in pathways related to degradation (13 out of top 20) (Fig. 1G).

Based on the 16S and ITS sequence data of 370 subjects, cross-kingdom association networks of the oral and fecal microbiota were built (Fig. S4). Both networks consisted of a similar number of connected ASVs (as nodes) (oral: 194; stool: 182); however, both the number of total interactions (as edges) (oral: 465; stool: 329) and the proportion of positive interactions (oral: 80.4%; stool: 66.9%) were higher in the oral network (Table S2). The oral network also had higher values of characteristic path length and clustering coefficient and a lower network heterogeneity than the fecal network.

### Oral–gut transmission of bacteria and fungi are limited in healthy adults.

**FIG 3:**
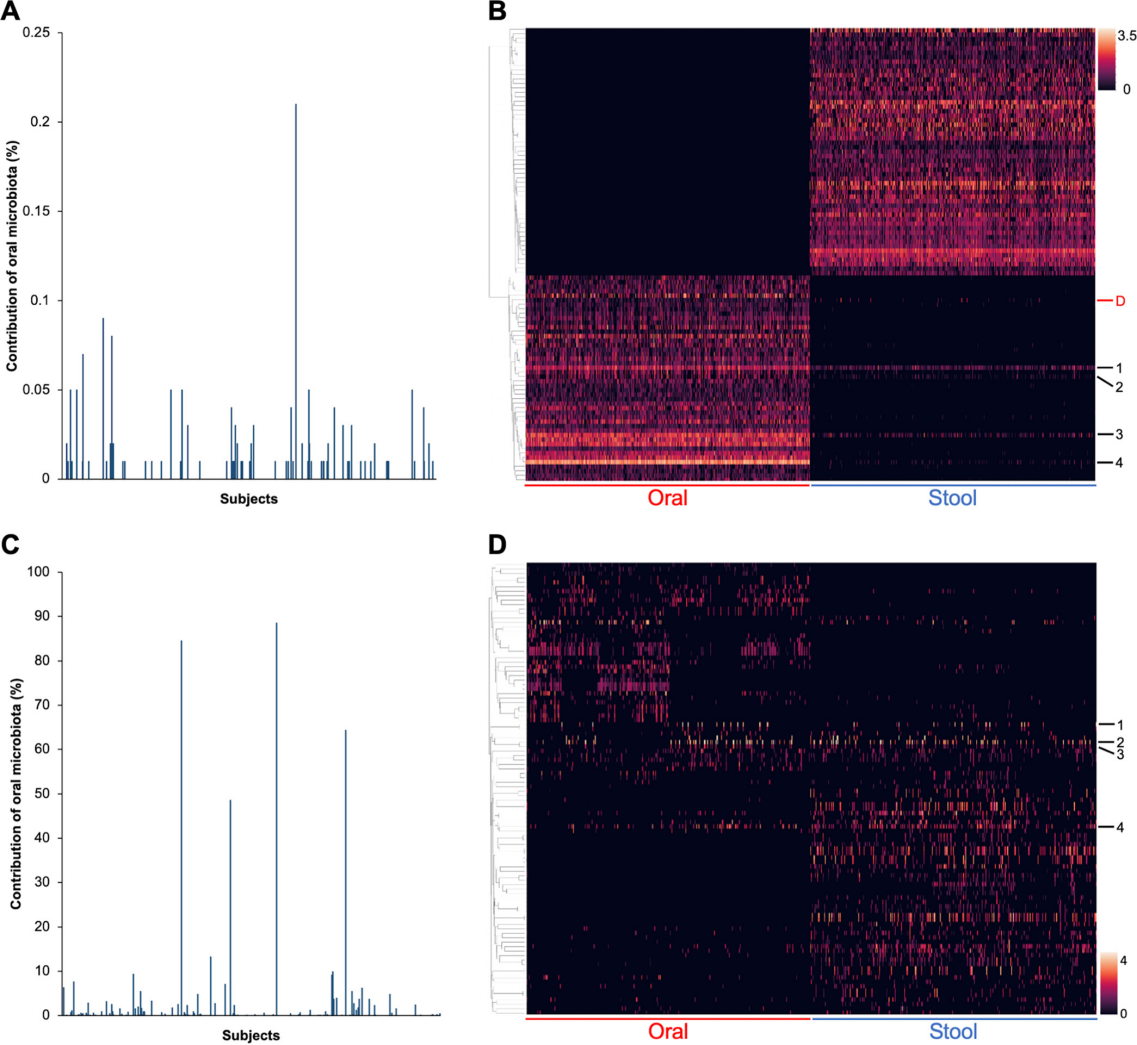
Limited oral–gut transmission of the bacterial (A, B) and fungal (C, D) microbiota in healthy Chinese adults. (A, C) Percent contribution of oral microbiota to stool microbiota per subject as estimated by SourceTracker2. *n* = 470 in panel A and *n* = 370 in panel C. (B, D) Heatmaps of the top 100 most prevalent ASVs across all samples. ASVs shared between paired oral and stool samples in > 10% (for bacteria) and > 3% (for fungi) of subjects are numbered in the heatmaps. Also labeled in panel B is an ASV belonging to Dialister invisus, which was previously shown to be common in paired oral and stool samples of Western populations. Refer to Tables 2 and S3 for details of the labeled ASVs.

The amount of oral–gut microbiota transmission was estimated using the Bayesian tool SourceTracker2. Results showed that > 99% of the subjects (*n* = 466) had a negligible oral-to-stool bacterial contribution of ≤ 0.05%, with 402 subjects (86%) showing no contribution at all (Fig. 3A). The highest contribution rate was observed to be 0.21%. A heatmap of the most prevalent bacterial ASVs across all samples showed that the majority of the prevalent bacterial taxa was distinct between the oral and fecal microbiota (Fig. 3B). Exceptions included four ASVs belonging to Streptococcus salivarius, Haemophilus parainfluenzae, Streptococcus sinensis, and Streptococcus sp., which were shared between oral and stool samples in 60.4%, 12.1%, 29.1%, and 13.2% of the subjects, respectively (Tables 2 and S3). Within subjects containing these ASVs in their paired oral and stool samples, a higher relative abundance in the oral than stool samples was observed both globally and in over 92% of the subjects for all the four ASVs. Notably, an ASV belonging to Dialister invisus, previously reported to be common in paired oral and stool samples of Western populations, was detected in both oral and stool samples of only 12 subjects (2.6%) in our cohort (7, 8).

**TABLE 2:** ASVs mostly shared between paired oral and stool samples^*a*^

		Taxonomy	Occurrence				Relative abundance in shared subjects		
Kingdom	Label^*b*^	BLAST (% identity)	Oral	Stool	Shared	EPS (%)^*c*^	Oral (%)	Stool (%)	Oral > stool
Bacteria	1	Streptococcus salivarius strain ATCC 7073 (100%)	451 (96.0%)	290 (61.7%)	284 (60.4%)		0.022	0.003	263 (92.6%)
	2	Streptococcus sinensis strain HKU4 (99.5%)	212 (45.1%)	83 (17.7%)	57 (12.1%)	8.0	0.014	0.001	54 (94.7%)
	3	Haemophilus parainfluenzae ATCC 33392 (100%)	445 (94.7%)	139 (29.6%)	137 (29.1%)		0.041	0.004	128 (93.4%)
	4	Multiple Streptococcus spp. (all 100%)	470 (100%)	62 (13.2%)	62 (13.2%)		0.259	0.001	62 (100%)
	D	Dialister invisus DSM 15470 strain E7.25 (100%)	250 (53.2%)	19 (4.0%)	12 (2.6%)	2.2	0.002	0.002	9 (75.0%)
Fungi	1	Candida albicans CBS 562 (100%)	20 (5.4%)	23 (6.2%)	14 (3.8%)	0.3	0.804	0.487	13 (92.9%)
	2	Candida albicans CBS 562 (99.5%)	51 (13.8%)	66 (17.8%)	31 (8.4%)	2.5	0.728	0.465	25 (80.6%)
	3	Candida albicans CBS 562 (99.5%)	32 (8.6%)	44 (11.9%)	17 (4.6%)	1.0	0.015	0.007	11 (64.7%)
	4	Candida parapsilosis ATCC 22019 (100%)	53 (14.3%)	113 (30.5%)	26 (7.0%)	4.4	0.354	0.078	20 (76.9%)

a All values are count (%) unless specified otherwise.

b Labels in heatmaps (Fig. 3).

c Expected probability of the ASV to occur in both oral and stool samples without assuming oral-gut transmission, calculated by multiplying the percent occurrence of “oral” by that of “stool”; not calculated for ASVs with an occurrence > 90% in either or both body sites.

For fungi, results of SourceTracker2 showed that > 96% of the subjects (*n* = 356) had a low oral-to-stool microbiota contribution of ≤ 5%, and 271 of them (73%) showed no contribution at all (Fig. 3C). Four subjects showed exceptionally high contributions of > 40%, and the highest contribution was estimated to be 88.5%. A heatmap of the most prevalent fungal ASVs across all samples showed that most fungal taxa were specific to the oral or fecal microbiota (Fig. 3D). Three ASVs belonging to Candida albicans and an ASV belonging to Candida parapsilosis were most shared between the oral and stool samples of the same subjects, with observed values of sharing between 3.8% and 7.0% (Tables 2 and S3). All these values were higher than the expected probability of finding the same ASVs in the two body sites by chance without assuming oral–gut transmission, as calculated by multiplying the percent occurrence of the ASVs in both body sites (8), with a difference reaching as high as 12.7-fold for one of the Candida albicans ASVs. Similar to the case of bacteria, within subjects containing these fungal ASVs in their paired oral and stool samples, a higher relative abundance in the oral than stool samples was observed both globally and in the majority (>64%) of the subjects for all the four ASVs. A few other fungal ASVs were present in both oral and stool samples (Fig. S5); however, they were only shared in < 2% of subjects, which were comparable to the expected probabilities without assuming oral–gut transmission (Table S4). Associating the degree of oral–stool sharing of the four potentially transmissible fungal ASVs with host variables revealed that sharing of the Candida albicans ASVs was significantly more common in subjects who are older, have a higher BMI, lack moderate–vigorous exercise, or with certain diet preferences (*P < *0.05); whereas that of the Candida parapsilosis ASV tended to be associated with a higher BMI (*P = *0.0555) (Table S5).

## DISCUSSION

Recent studies have suggested the presence of an oral–gut bacterial microbiota axis in gastrointestinal diseases (1, 2). In particular, the oral species Fusobacterium nucleatum has been repeatedly reported to be enriched in the gut microbiota of colorectal cancer patients from multiple cohorts (1). However, whether a similar axis exists in healthy individuals is still in debate (5, 8). Based on one of the largest cohorts available to date for studying the oral–gut microbiota axis in health, here we examined the extent of oral–gut microbiota transmission at the ASV level and tested the hypothesis that there is limited oral–gut transmission of both the bacterial and fungal microbiota in healthy Chinese adults. Our work is novel with a focus on a non-Western population and represents the first attempt to examine oral–gut transmission of the fungal microbiota, a clinically relevant yet understudied component of the human microbiome, based on a large cohort.

We showed that both the bacterial and fungal microbiota in the oral cavity and gut of healthy subjects differed in terms of alpha and beta diversity. While our finding on the bacterial microbiota agrees with previous studies, new observations on the fungal microbiota further indicate that the oral cavity and gut of humans harbor distinct microbial communities from multiple biological kingdoms (4, 5, 7, 8). Besides, our network analysis revealed a higher proportion of positive interactions, higher values of characteristic path length and clustering coefficient, and a lower network heterogeneity in the oral network than the fecal network, suggesting more cross-feeding/co-colonization, a lower navigability, a higher modularity, and a lower tendency to form hubs of the oral microbiota, respectively (11). Collectively, our findings indicate that the oral and gut microbiomes are distinct with regard to community composition, function and structure.

Bayesian analysis revealed a negligible oral-to-stool bacterial contribution. Subsequent detailed comparisons of individual prevalent bacterial ASVs between paired oral and stool samples provided further evidence to a limited overlap between the oral and gut bacterial microbiota. ASVs belonging to Streptococcus salivarius, Haemophilus parainfluenzae and Streptococcus sinensis were among the most shared between the oral and stool samples of our subjects. Streptococcus salivarius and Haemophilus parainfluenzae, alongside seven other bacterial species, were also detected in both saliva and stool samples of a U.S. population (7). Streptococcus sinensis, first isolated from a patient with infective endocarditis in Hong Kong, is a commensal of the oral cavity which appears to be prevalent only in Southeast Asia, in particular Hong Kong (12). The high prevalence of these ASVs in the paired oral and stool samples, together with the higher relative abundance of them in the oral compared to stool samples among subjects containing them in both body sites, suggest potential oral-to-gut transmission and subsequent colonization of them in the gut (5, 7).

Dialister invisus has been repeatedly reported in paired oral and stool samples of Western populations, albeit usually in low relative abundance (typically <1%) (7, 8). For instance, Rashidi et al. detected a *D. invisus* ASV in 59% and 33% of the salivary and stool samples, respectively, of 66 healthy adults from the United Kingdom and Sweden, which was shared in 24.2% of the subjects (8). A *D. invisus* ASV was also detected in over 53% of the oral rinse samples in our Chinese cohort (relative abundance: 0.15%); however, it was only detected in 4% of the stool samples (relative abundance: 0.41%) and shared between paired oral and stool samples in 2.6% of the subjects, a value consistent with the probability expected without assuming oral–gut transmission. The lower prevalence of the ASV in our stool samples is unlikely a result of insufficient sequencing depth since the mean number of quality-filtered reads is comparable between the two studies (Rashidi et al.: 9,146; this study: 7,305). It is also unlikely to be due to inefficient DNA extraction since the DNeasy PowerSoil kit (formerly MO BIO PowerSoil DNA isolation kit) we used here has comparable, if not better, performance to the Human Microbiome Project protocol in the recovery of the human gut microbiota (13). Overall, the consistency observed between the prevalence of the Dialister invisus ASV in paired oral and stool samples and the probability expected without assuming oral–gut transmission, together with its comparable relative abundance between paired oral and stool samples, suggest that the ability of Dialister invisus to transit from the mouth and colonize the gut is population-specific, which may be attributed to factors such as host genetics and diet. It also highlights the merit of our current work in providing data from a non-Western population.

Similar to the case of the bacterial microbiota, Bayesian analysis also revealed a low oral-to-stool fungal microbiota contribution. For those four subjects who showed exceptionally high contributions, we examined the host variables in detail but detected no peculiar variables that might explain the extreme high values. We speculate that the high values of contribution observed in these few subjects may be related to the low complexity of the oral and gut fungal microbiota, comprising as few as two ASVs in one of the subjects concerned, which could lead to large changes in the calculation even if only one ASV is altered. Detailed comparisons of individual prevalent fungal ASVs between paired oral and stool samples showed that most fungal taxa were specific to the oral or fecal microbiota. Notable exceptions included three ASVs belonging to Candida albicans and an ASV belonging to Candida parapsilosis. The observed values of sharing for all these fungal ASVs were higher than the expected probability without assuming oral–gut transmission, with a difference reaching as high as 12.7-fold for one of the Candida albicans ASVs. Besides, their relative abundance was higher in the oral than stool samples among subjects containing them in both body sites. Collectively, these results suggest that these *Candida* ASVs do not simply co-occur in the oral cavity and gut but are actually transmissible between the two body sites. Indeed, Candida albicans has been shown to be able to optimize its growth and metabolism in according to various stress, such as pH variation and oxygen availability, thereby allowing it to adapt to different niches of the gastrointestinal tract (14). However, further studies are needed to understand the exact mechanisms underlying its potential transmission between the oral cavity and gut.

Since the oral–gut transmissibility of the four fungal ASVs was supported by multiple evidence, we then associated the degree of oral–stool sharing with a list of host variables for each of the ASVs to understand if their degree of transmissibility is related to any of the host factors. Results showed that sharing of the Candida albicans ASVs was significantly more common in subjects who are older, have a higher BMI, lack moderate–vigorous exercise, or with certain diet preferences, whereas that of the Candida parapsilosis ASV tended to be associated with a higher BMI. A previous study on the changes in the gut microbiome from newborn to centenarian of a healthy Japanese population has reported enrichments of oral bacteria such as *Porphyromonas* and *Fusobacterium* in elderly-associated coabundance groups, clusters of correlated genera across age groups, suggesting that oral–gut transmission is increased in the elderly (15). Indeed, aging and obesity have been reported to increase the permeability of the intestinal barrier (16, 17). Besides, although acute exercise increases intestinal permeability, accumulating evidence has suggested that chronic exercise actually improves gut barrier integrity (18). Moreover, diet is also an important factor that can alter intestinal permeability (19). Therefore, we speculate that an advanced age, a high BMI, lack of moderate–vigorous exercise, or certain diet preferences increased intestinal permeability of the subjects, thereby allowing microbial cells to leak across the intestinal barrier into the gut lumen.

There are several limitations to our study. First, DNA was extracted from the oral rinse and stool samples using two different commercial DNA extraction kits. The use of different extraction protocols can lead to potential inherent biases. However, as both kits involve a bead beating step with the same PowerBeads for mechanical lysis of cells and multiple studies have reported a minimal effect of the use of different DNA extraction methods on the recovery of the human oral microbiota, we expect that any potential extraction biases introduced and thus their effects on the current analysis would be small (20, 21). Nonetheless, extraction effects should be considered a potential confounding factor in interpreting the results obtained here. Second, most metadata collected in this study were self-reported by the subjects. Third, DNA but not viable cells was used to indicate the presence/absence of microbes. Lastly, our findings do not reveal the exact oral–gut transmission routes.

To conclude, we uniquely characterized here the bacterial and fungal microbiomes in paired oral and stool samples from a large non-Western population and showed that oral–gut microbiota transmission in healthy adults is limited across biological kingdoms. Exceptions included a few bacteria belonging to the genera Streptococcus and Haemophilus and a few fungal members belonging to the *Candida* genus, the transmission rate of the latter is associated with a few host factors, including an older age and a higher body mass index. Our findings have practical implications that detection of a large amount of shared bacterial or fungal members between the two body sites within the same subject as an indicator of potential medical conditions.

## MATERIALS AND METHODS

### Study population.

Subjects were recruited from the Hong Kong public from November 2017 to August 2018 as part of the HKGutMicMap study of the local healthy population (22). Each participant completed a questionnaire, including information on sociodemographic characteristics, lifestyle and diet. Anthropometric parameters such as body weight and height were measured by research staff at the time of recruitment. The inclusion criteria for the current analysis were ethnically Chinese aged ≥ 18 years at the time of recruitment. Subjects with a history of intestinal polyp/malignant tumor, inflammatory bowel disease or irritable bowel syndrome, intestinal resection, major chronic diseases or antibiotics use within last 3 months were excluded. This study was approved by the Joint Chinese University of Hong Kong–New Territories East Cluster Clinical Research Ethics Committee (CREC 2016.707). Written informed consent was obtained from all participants prior to sample collection.

### Sample collection, DNA extraction, and amplicon sequencing.

Oral rinse samples were collected in 20 mL 0.9% normal saline gargled twice, for 20 and 10 s. One mL of the oral rinse solution was then centrifuged at 5,000 g for 5 min and DNA extracted from the pellet using the QIAamp BiOstic Bacteremia DNA kit (Qiagen), optimized for low-bacterial-biomass samples, following the manufacturer’s instructions. Stool samples were collected by the subjects using stool collection kits provided, delivered to the laboratory within 2 h of defecation, and stored at −80°C until further processing. DNA was extracted from 0.1 g of homogenized stool using DNeasy PowerSoil kit (Qiagen) following the manufacturer’s instructions. The V3–V4 region of the bacterial 16S rRNA gene was amplified from the oral and stool samples using universal primers 341F (5′-CCT ACG GGN GGC WGC AG-3′) and 806RB (5′-GGA CTA CNV GGG TWT CTA AT-3′), whereas ITS1F (5′-CTT GGT CAT TTA GAG GAA GTA A-3′) and ITS2 (5′-GCT GCG TTC ATC GAT GC-3′) were used to amplify the fungal ITS1 region. PCR products were pooled and sequenced on an Illumina MiSeq instrument (Illumina) at The Genomics Core Facility of the Weill Cornell Medicine Core Laboratories Centre following the 2 × 300 bp paired-end sequencing protocol. Negative controls (distilled water as the template), positive controls (ZymoBIOMICS Microbial Community DNA Standard [Zymo Research]), and technical replicates (randomly selected DNA samples) were also amplified and sequenced for quality control.

### Microbiome analysis.

Microbiome analysis was performed with QIIME2 2020.11 as previously described (23, 24). In brief, primers were trimmed from demultiplexed raw sequence data using q2-cutadapt, without allowing for insertions or deletions during primer matching. Paired-end reads were then quality-filtered, joined and denoised using q2-dada2 (25). ASVs were generated using Naïve Bayes classifiers trained on the V3–V4 region of the SILVA 138 SSU Ref NR 99 data set for 16S and full-length UNITE v8.3 dynamic data set for ITS with q2-feature-classifier (26, 27). ASVs with < 10 total reads or present in < 2 samples were removed. Mitochondrial and chloroplast reads were also discarded. Samples with < 1,000 or < 200 quality-filtered sequence reads were removed from the 16S and ITS data sets, respectively.

Alpha and beta diversity analysis, principal coordinates analysis (PCoA), and effect size analysis were performed using q2-diversity after rarefying the samples to the smallest number of reads. Alpha diversity metrics computed included the number of observed ASVs and Shannon diversity, whereas presence/absence-based Jaccard distance was used for beta diversity estimation. Effect size of metadata variable was calculated based on Bray–Curtis dissimilarity using the adonis function with 9,999 permutations. Alpha rarefaction curves were generated using q2-diversity before removal of rare ASVs. Heatmaps of the most prevalent ASVs were generated using the heatmap function of q2-feature-table.

### Prediction of functional potential.

Metabolic functions were predicted from the 16S data using q2-picrust2 in QIIME2 2019.7 (28). A PCoA plot was then generated as described above. Differentially abundant MetaCyc pathways were identified using the compositionality-aware q2-Songbird plugin (29).

### Source tracking.

The degree of contribution of the oral microbiota to the stool microbiota per subject was estimated using a Bayesian approach, namely, SourceTracker2 (9). Originally designed to estimate the proportion of microbial contaminants in a sink sample that come from possible source environments, SourceTracker has been established for effective tracking 16S rRNA sequences among body sites of the same individuals (30). Here, each oral sample was set as an individual source, whereas individual stool samples were set as sinks.

### Network analysis.

Cross-kingdom association networks were built from samples with both 16S and ITS sequence data as previously described (24). In brief, rare bacterial ASVs present in < 20% of samples and fungal ASVs in < 5% of samples were filtered. Cross-kingdom networks were then constructed using the R package SpiecEasi ver. 1.1.1 with the Meinshausen–Bühlmann neighborhood selection method (31). Network parameters were calculated using NetworkAnalyzer in Cytoscape 3.8.0 (32).

### Statistical analysis.

Differences in alpha diversity between groups were tested using Kruskal–Wallis test, whereas differences in beta diversity were tested using permutational multivariate analysis of variance (PERMANOVA) with 9,999 permutations using the adonis function in q2-diversity. In effect size analysis, *P* values were adjusted using the Benjamini–Hochberg procedure to control for multiple comparisons. In association analysis between host variables and the degree of oral–gut microbiota transmission, categorical variables were compared using Fisher’s exact test, whereas continuous variables were tested using Welch’s *t* test. Differences were considered statistically significant when *P < *0.05 or false discovery rate (FDR) < 0.1.

### Data availability.

All sequence reads generated in this study have been deposited to the NCBI Sequence Read Archive (SRA) under Bioproject accession PRJNA834584.
