# Supplementary material for: Extent of Oral–Gut Transmission of Bacterial and Fungal Microbiota in Healthy Chinese Adults
Source: Microbiol Spectr. 2023 Jan 10;11(1):e02814-22. doi: 10.1128/spectrum.02814-22 (PMC9927295; doi:10.1128/spectrum.02814-22)
Supplement: Supplemental file 1 — Fig. S1 to S5. Download spectrum.02814-22-s0001.pdf, PDF file, 4.7 MB [file spectrum.02814-22-s0001.pdf]

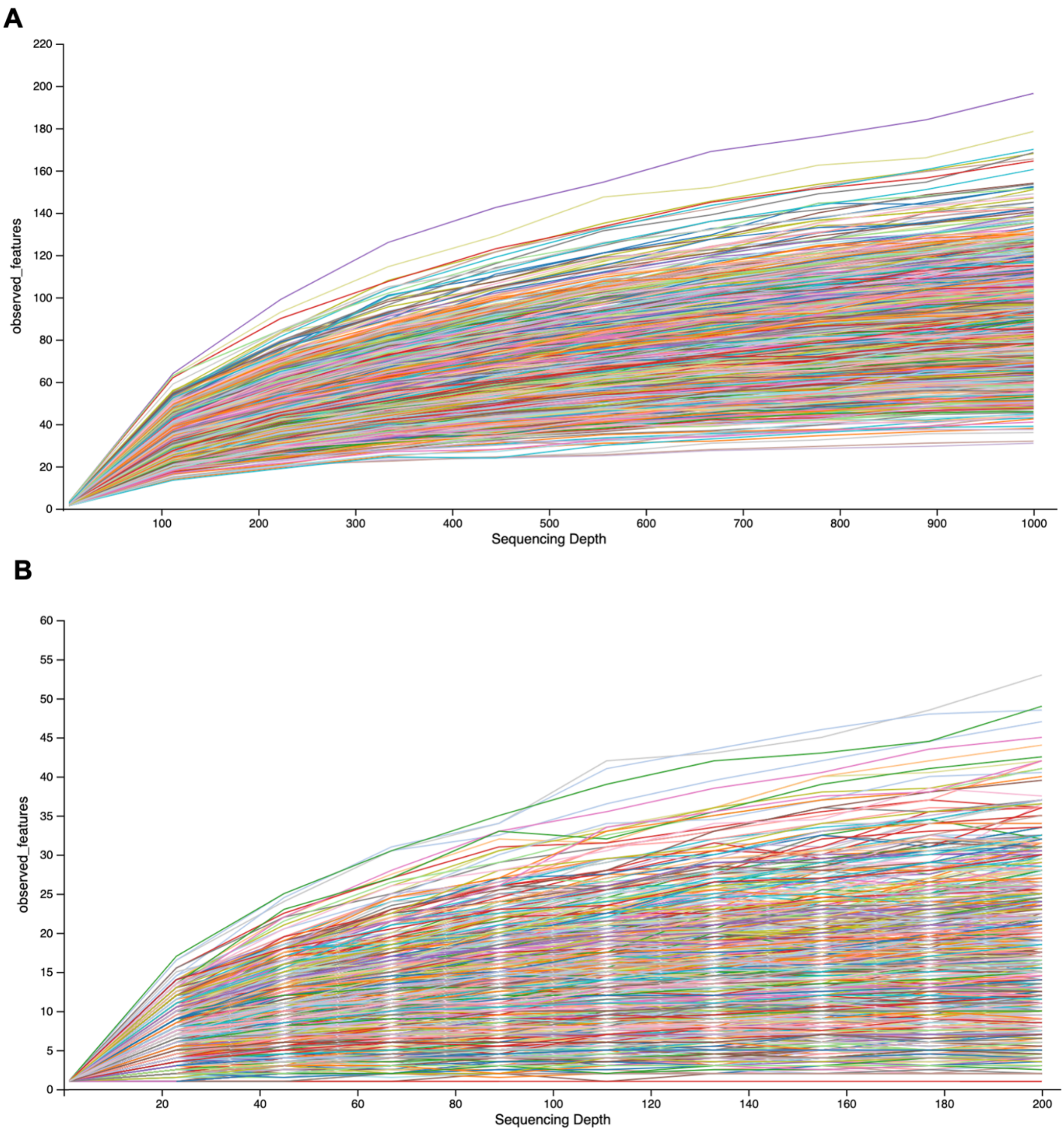

FIG S1 Rarefaction curves of the bacterial (*A*) and fungal (*B*) microbiomes at sequencing depths of 1,000 and 200 reads, respectively.

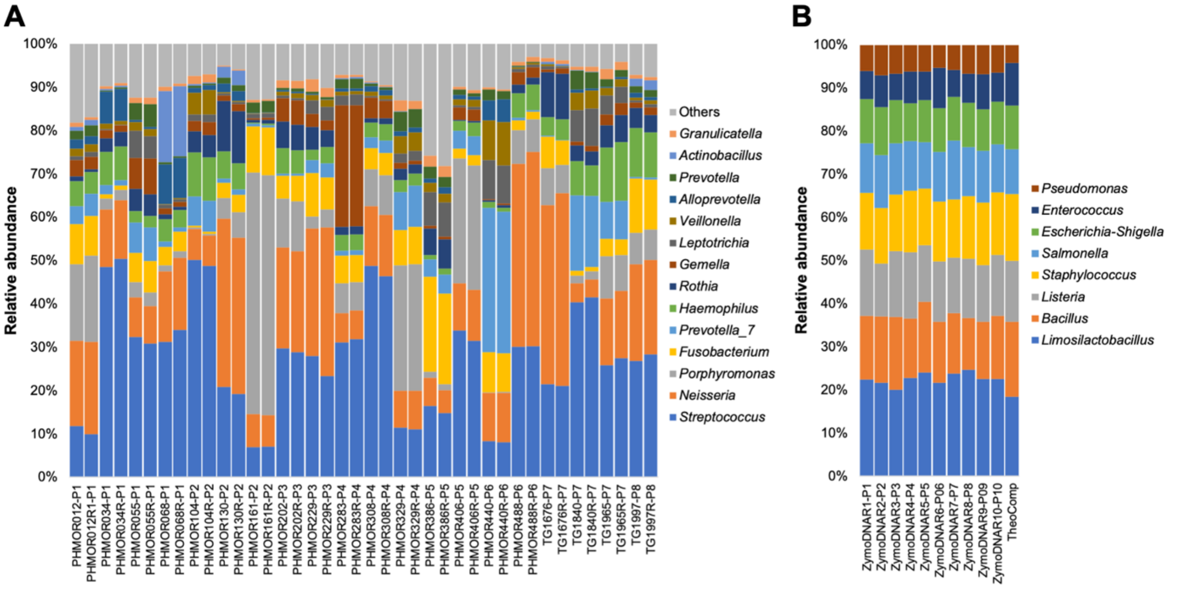

C

Kingdom	Sample ID	Raw reads	Denoised reads	Taxonomy
Bacteria	Neg071	16	0	-
	Neg072	8	0	-
	Neg073	7	0	-
	Neg074	11	0	-
	Neg075	9	0	-
	Neg076	11	0	-
	Neg077	6	0	-
	Neg078	8	0	-
	Neg079	13	0	-
	Neg080	11	0	-
Fungi	NAi1301	5	0	-
	NAi1302	7	0	-
	NAi1303	14	0	-
	NAi1304	4	0	-
	NAi1305	5	0	-
	NAi1306	15	1	*Auricularia auricula-judae*
	NAi1307	12	0	-

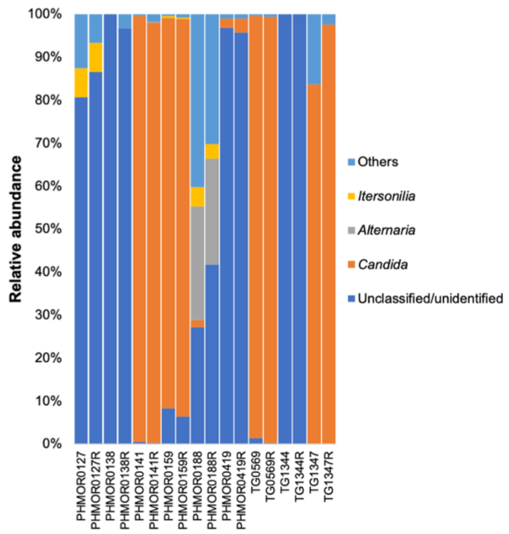

FIG S2 Results of quality control for bacterial (A–C) and fungal (C–D) microbiome analysis. (A) Taxonomic barplots of major bacterial genera in technical replicates (n = 2) of oral rinse DNA samples from 20 randomly selected subjects. Replicates are denoted with a “R” in the sample IDs. Taxa with a mean relative abundance <1% were grouped into “Others”. (B) Taxonomic barplots of bacterial genera in microbial community DNA standards sequenced (n = 10). A bar representing the theoretical composition was also included to the far right for reference. (C) Table summarizing the number of raw and denoised reads in negative control samples (distilled water) for bacterial (n = 10) and fungal (n = 7) microbiome analysis. For reads passing the denoising step, their taxonomic identity was also provided. (D) Taxonomic barplots of major fungal genera in technical replicates (n = 2) of oral rinse DNA samples from nine randomly selected subjects. Replicates are denoted with a “R” in the sample IDs. Taxa with a mean relative abundance <1% were grouped into “Others”.

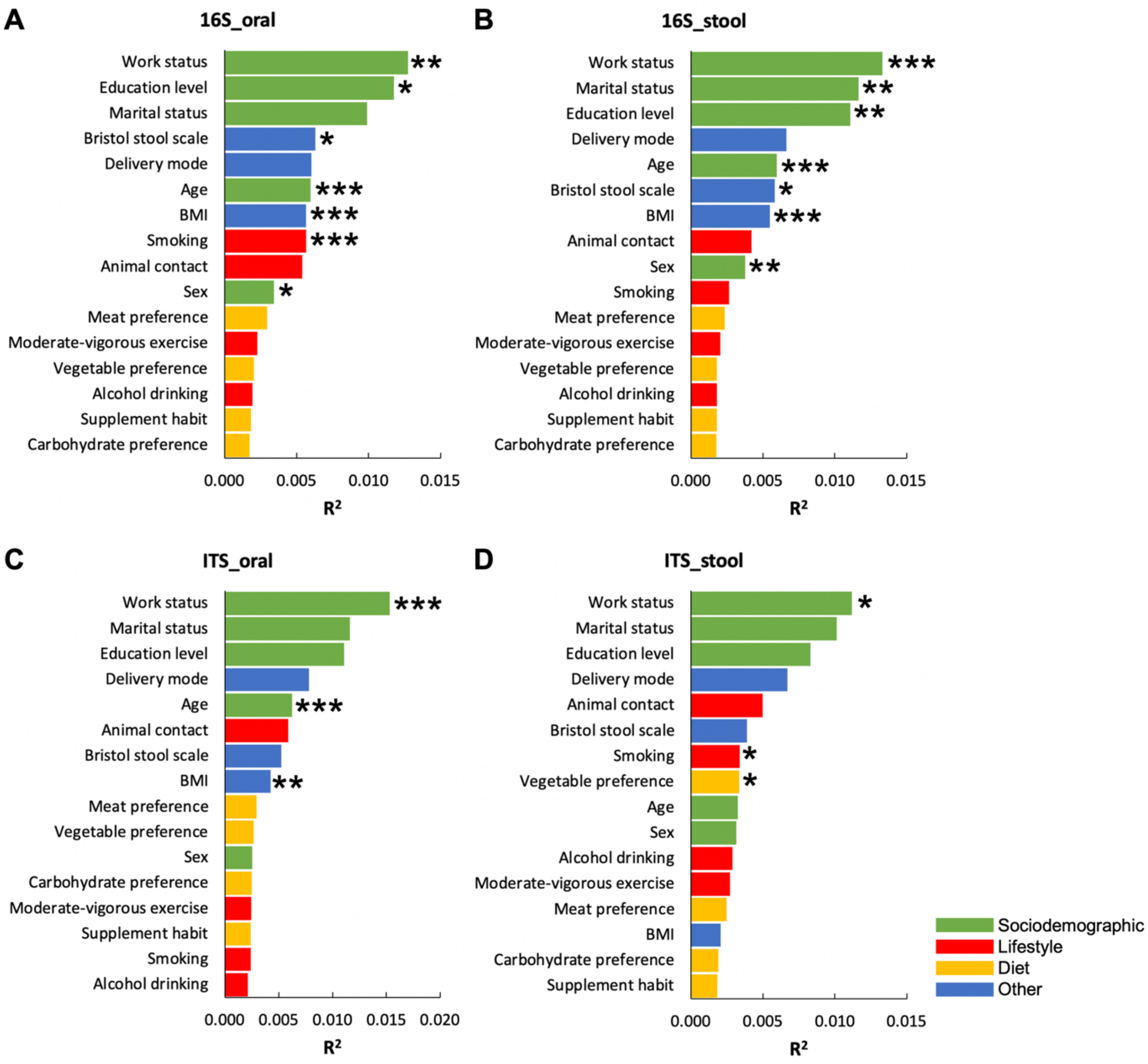

FIG S3 Effect size of metadata variables on the bacterial (A–B) and fungal (C–D) microbiota composition in oral (A, C) and stool samples (B, D) based on Bray–Curtis dissimilarity. Bars are colored according to the category of the variables. *, $P < 0.05$; **, $P < 0.01$; ***, $P < 0.001$. All variables remained significant after controlling for multiple comparisons (FDR < 0.1), except for variables in panel D.

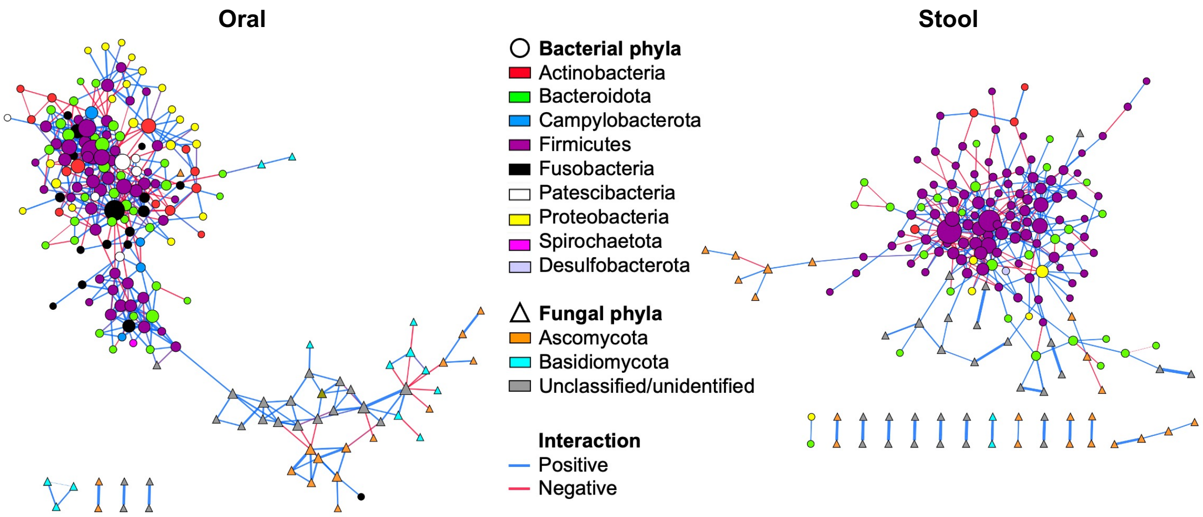

FIG S4 Cross-kingdom association networks of the oral and gut microbiomes of healthy Chinese adults constructed using SPIEC-EASI. Taxa, as nodes, from different kingdoms are in different shapes and those from different phyla are in different colors. The sizes of nodes are proportional to the node degree. Edges in blue represent positive interactions, whereas those in red are negative interactions. The thickness of edges are proportional to the interaction strength. Only connected nodes are shown here.

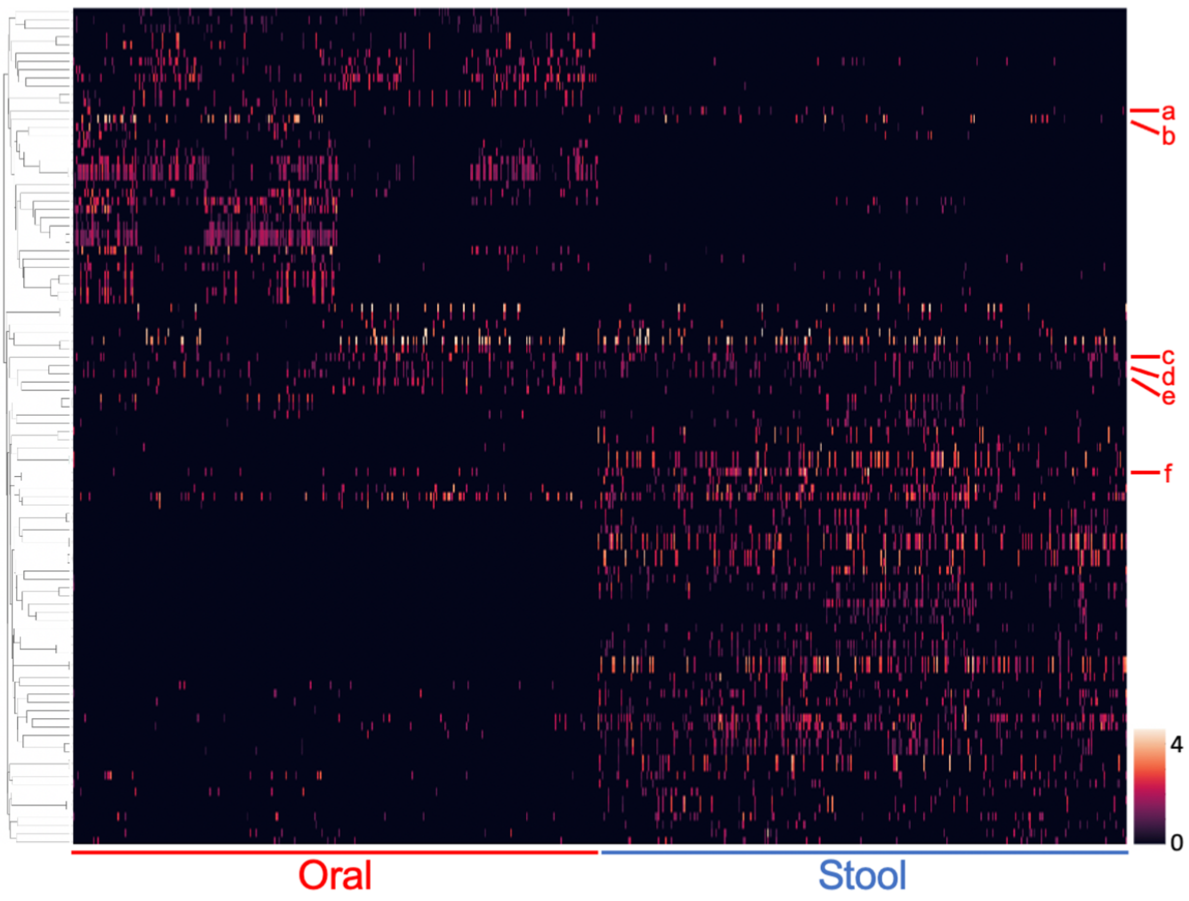

FIG S5 Heatmap of the top 100 most prevalent fungal ASVs across all samples. ASVs detected in both oral and stool samples of >10 individuals but shared between <10% of paired samples are labelled in lowercase. Refer to Table S3 for details of the labelled ASVs.
